# Single use flexible ureteroscopes: Current status and future directions

**DOI:** 10.1002/bco2.265

**Published:** 2023-07-05

**Authors:** Patrick Juliebø‐Jones, Eugenio Ventimiglia, Bhaskar K. Somani, Mathias Sørstrand Æsøy, Peder Gjengstø, Christian Beisland, Øyvind Ulvik

**Affiliations:** ^1^ Department of Urology Haukeland University Hospital Bergen Norway; ^2^ Department of Clinical Medicine University of Bergen Bergen Norway; ^3^ EAU YAU Urolithiasis group Armhem Netherlands; ^4^ Department of Urology IRCCS Ospedale San Raffaele Milan Italy; ^5^ Department of Urology University Hospital Southampton UK

**Keywords:** disposable, ureteroscopy, urinary calculi, urolithiasis

## Abstract

**Introduction:**

Single use ureteroscopes are a technological innovation that have become available in the past decade and gained increased popularity. To this end, there are now an increasing number of both benchside and clinical studies reporting outcomes associated with their use. Our aim was to deliver a narrative review in order to provide an overview of this new technology.

**Methods:**

A narrative review was performed to gain overview of the history of the technology's development, equipment specifications and to highlight potential advantages and disadvantages.

**Results:**

Findings from preclinical studies highlight potenial advantages in terms of the design of single use ureteroscopes such as the lower weight and more recent modifications such as pressure control. However, concerns regarding plastic waste and environmental impact still remain unanswered. Clinical studies reveal them to have a non inferior status for outcomes such as stone free rate. However, the volume of evidence, especially in terms of randomised trials remains limited. From a cost perspective, study conclusions are still conflicting and centres are recommended to perform their own micro cost analyses.

**Conclusions:**

Most clinical outcomes for single use ureteroscopes currently match those achieved by reusable ureteroscopes but the data pool is still limited. Areas of continued debate include their environmental impact and cost efficiency.

## 1 INTRODUCTION

The lifetime prevalence of kidney stone disease is approximately 12% in Europe, and it represents a large burden on health systems.^1^ The triad of shockwave lithotripsy (SWL), percutaneous nephrolithotomy (PCNL) and ureteroscopy (URS) represent the core treatments available when intervention is required.^2^ Over the past 20 years, the latter has emerged as an increasingly used modality and now represents a preferred option for many stone scenarios.^3^ This is largely because of the plethora of technical advancements that have taken place in this field. This includes the introduction of digital technology, optic systems and a wide selection of accessories that can be used.^4^, ^5^ As such, the selection of patient groups that can be safely treated has been expanded and now includes patients at the extremes of age, pregnancy and complex anatomy such as renal transplants.^6^, ^7^, ^8^ Single use (SU) flexible ureteroscopes (also referred to as ‘disposable’) are another development that became commercially available in October 2015.^9^ Their invention was largely borne out of attempts to eliminate maintenance requirements and the associated costs as well as durability issues such as deflection loss after multiple usages.^10^ Since then, they have become subject to increasing attention and a growing body of research is now available in the form of both benchtop and clinical studies. At present, their role in clinical practice receives no recommendation from international guidelines and with the rapid dissemination of experimental research, it can be a challenge for clinicians to make an assessment and know if reality meets expectations. Our aim was to perform a review of this novel piece of equipment and provide an overview of its current status in clinical practice.

## 2 MATERIALS AND METHODS

A comprehensive search of literature was performed to identify studies on SU ureteroscopes. All study types were eligible for inclusion. Bibliographic databases searched included PubMed/MEDLINE, Google scholar and Scopus. Reference lists and relevant grey literature such as conference abstracts were also searched. Search terms included ‘single use’, ‘disposable’, ‘ureteroscopy’, ‘retrograde intra‐renal surgery’ and ‘minimally invasive surgery’. The results have been summarised in a narrative format the following key areas identified: history and development, equipment specifications, equipment properties/findings from clinical studies, cost, environmental impact and future perspectives.

## 3 HISTORY AND DEVELOPMENT

**FIGURE 1:**
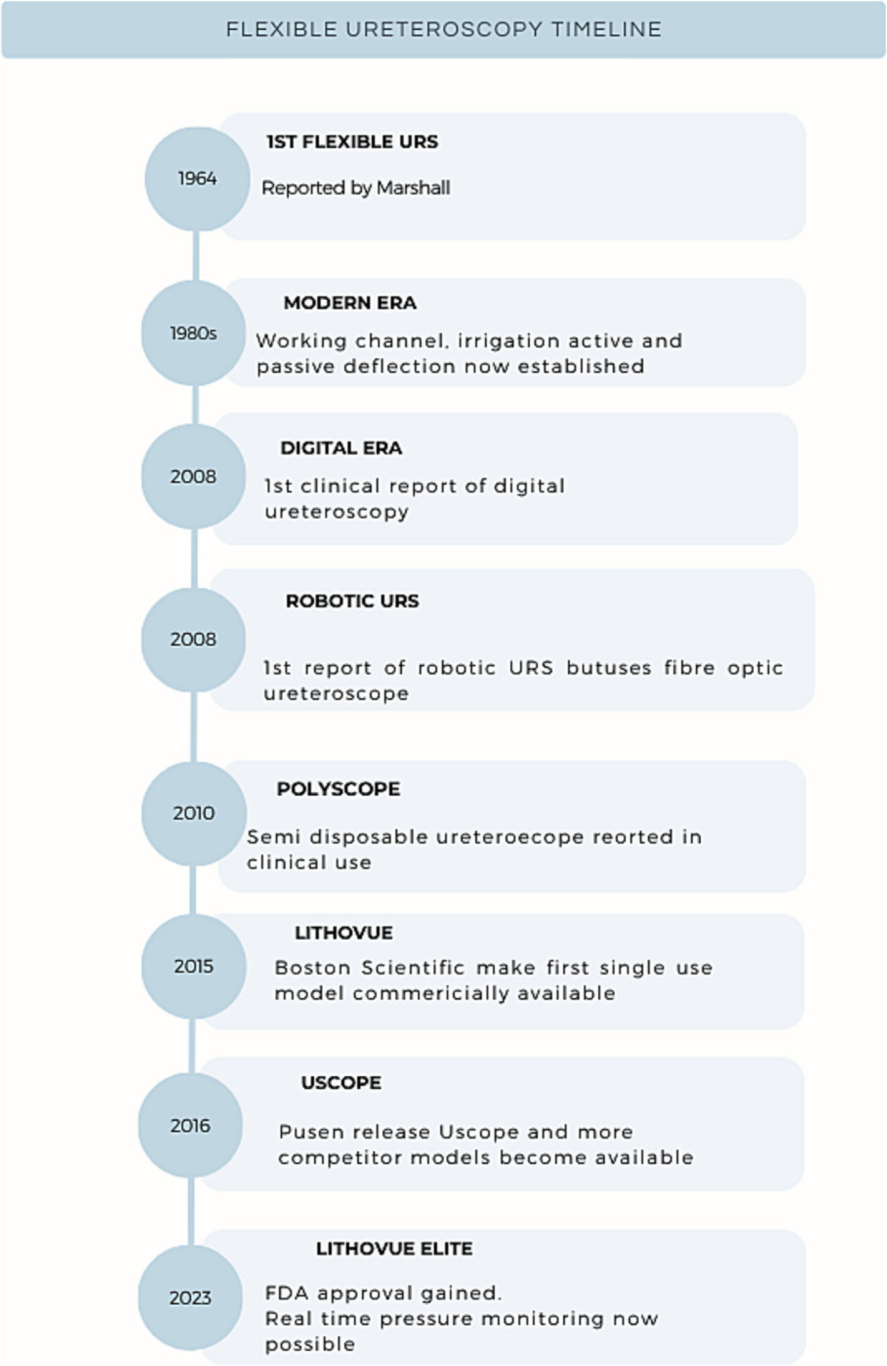
Historical timeline of innovations in ureteroscopy.

One of the earliest reports of flexible URS was by Marshall et al. in 1964 where an impacted ureteric calculus was visualised.^11^ Four years later, Tagachi performed the first diagnostic flexible URS with unilateral active deflection.^5^, ^12^ Later in 1987, Aso et al. performed therapeutic removal of upper ureteral and renal stones in 21 patients.^13^ By the late 1980s, working channels and irrigation were integrated but major complication rates were relatively high (Figure 1). In a series of 125 URS procedures reported by Wickham in 1987, 3% required ureteric re‐implantation.^14^ Fibre‐optic technology was the only option until 2008, when the first operative experiences with digital imaging was reported in a series of eight patients by Humphreys et al.^15^ That model was the DUR‐D™ made by Gyrus ACMI, a company that was taken over by Olympus in 2008. Digital ureteroscopes house the imaging chip at the distal end (often referred to as ‘chip on tip’) and removes the inconvenience of a camera head attachment as well as the white light cable. In October 2015, Boston Scientific (Marlborough, MA) introduced the first commercially available SU ureteroscope (LithoVue™) into clinical practice. Pusen (Zhuhai Pusen Medical Technology Co., Ltd., Zhuhai, China) introduced the next model (Uscope® UE3011 S) and, within 12 months, several other companies had released other versions.^16^ Prior to this, there had been attempts with experimental designs to introduce elements of SU equipment to the ureteroscope, in what was described as ‘semi‐disposable’ technology. An example was the PolyScope™ (PolyDiagnost GmbH, Pfaffenhofen, Germany), which gained FDA approval in 2009 and was first described in the clinical setting by Bader et al., which incorporated use of combining a re‐usable fibre‐optic bundle with a disposable multi‐lumen catheters (outer size 8 Fr) and steering system.^17^, ^18^, ^19^ However, clinical results were inferior to reusable (RU) ureteroscopes and therefore uptake was limited.^20^

## 4 EQUIPMENT SPECIFICATIONS

The lighting system is LED in the majority of the models with source integrated in the handle, but there are few exceptions such as Shaogang that has an external fibre optic cable attachment.^21^ The latter is a disadvantage as this additional cable adds to weight and can restrict movement. In contrast to many of the RU models, SU models often have two exit points for the light at the tip. For the camera sensor type, nearly all models used the complementary metal oxide semiconductors (CMOS) as opposed to alternatives such as charge‐coupled devices (CCDs). The former are cheaper to produce, use less energy, have faster processing and generate less heat.^22^ Regarding the ‘body mass index’ of the ureteroscopes, a term coined by Proietti et al., values are lower than RU counterparts.^23^ The lightest model is Neoscope (119 g) and the heaviest alternative is the Olympus URF V2 that weighs 942 g.^24^ Nearly all models have a 3.6 Fr working channel except for models such as the Indoscope (BioradMedisys™, Pune, India) (3.3 Fr) and the RP‐U‐C12 (REDPINE Medical Instruments, Guangzhou, China) (3.2 Fr). All SU models have a single working channel in contrast to certain RU models that have dual channels such as the Cobra Vision™ (Richard Wolf, Knittlingen, Germany), which houses 2 × 3.3 Fr lumens. Note the latter is disadvantaged by having to compensate with larger outer scope diameter at 9.5 Fr.^21^ Bidirectional deflection of 270° in most models. The maximum recorded deflection in a study is 300° with the Dornier AXIS™ (Webling, Germany).^25^ The latter ureteroscope also has the first version that is purposely built for use in females and is shorter (45 cm compared to 65 cm).^26^ However, this is not yet commercially available. Earlier models had a tip and shaft that were uniform in size but now tapered tip versions are also available. The smallest tip currently available is the WiScope (7.4 Fr). Overview of specifications of different models is provided in Table 1. All models are compatible with robotic platforms for URS.^27^ Purchase prices range from US$700 to US$3180.^20^

**TABLE 1:** Comparison of characteristics between single use ureteroscopes and digital reusable ureteroscopes.

Scope (manufacturer)	Deflection up/down (°)	Weight (g)	Working length (cm)	Shaft/tip diameter (Fr)	Working channel exit	Acquisition cost (USD)*
Digital reusable ureteroscopes						
Flex‐XC	270/270	562	67	8.5/7.9	3 o'clock	22 451
URF V3	275/275	940	98	8.4/8.5	9 o'clock	13 488–26 977
Single use ureteroscopes						
LithoVue	270/270	277	65	9.5/7.7	3 o'clock	1300
Uscope PU3033A	270/270	180	65	7.5/7.5	3 o'clock	800
Dornier Axis	275/275	160	66	8.5/8.5	3 o'clock	1148
Neoflex	280/280	147	68	9/9	3 o'clock	750
Flex‐X^C1^	270/270	104	70	8/8	3 o'clock	787
WiScope	275/275	185	67	8.6/7.4	12 o'clock	740

* Prices vary depending on country of purchase.

### 4.1 Additional features of newer generation models

Within a short time period, numerous modifications have been introduced. Most models are now available with the option of the articulated lever executing either standard or reverse (also referred to as contra‐positive or European style) deflection according to preference. Similarly, some models such as WiScope are available in left‐ and right‐handed versions. Certain models now have an autolock function that can be applied at the surgeon's discretion, for example, once deflected in lower pole. Such ergonomic improvements are welcomed given that 39% of endourologists have been reported to experience orthopaedic problems in their hand and/or wrist.^21^ Randomised trial reported by Ali et al. found that SU ureteroscope (WiScope®) resulted in significantly less limb fatigue.^28^ Initial models required purchase of proprietary monitor and processor. Newer SU models have different connector types; for example, USB and cam allow for attachment to existing monitors, thus improving compatibility with existing equipment.^29^ Another recent update is the LithoVue™ Empower, which has an built–in basket, which the surgeon can control themselves. Another model from the same company is the LithoVue Elite™, which can perform real time monitoring of intra‐renal pressure. This gained US Food and Drug Agency (FDA) approval in February 2023, and although there are no published studies to date, a prospective clinical study (NCT05201456) is currently in progress across 11 global sites.^30^

Of note too, is the development of SU semi‐rigid ureteroscopes such as the RIWO D‐URS™ (Richard Wolf GmbH, Knittlingen, Germany), which has a hybrid function in that the tip is flexible. It has an outer diameter of 9 Fr, and a special feature is that it houses three channels consisting of an outflow channel, a working channel (3.6 F) for accessories and a dedicated channel for the laser fibre (1.6 Fr). To date, formal studies are lacking, which report its use in a clinical setting.

### 4.2 Equipment properties—Findings from benchside studies

Essential scope features include manoeuvrability, optical characteristics (image resolution, colour perception and luminosity), working channel flow, deflection (including when working channel occupied) and durability (i.e., need to maintain function over the whole case). A number of in vitro simulation studies have been performed assessing these properties. Dragos et al. compared four different SU models with four of the main RU models in use.^21^ This found deflection to be superior in SU models, but these were associated with more deflection loss after use. While irrigation flow was superior in SU models, image properties were inferior. So et al. evaluated surgeon preferences when using a range of SU models as well as assessments at completing tasks, for example, repositioning lower pole stone in a model.^31^ Although certain models received higher subjective ratings, these did not translate into higher performance scores when measured objectively. One of the limitations in studies comparing ureteroscopes is the heterogeneity of the assessments and methods employed for measurements. New aids such as the The Uniform grading tooL for flexIble ureterorenoscoPes (TULIP‐tool) will aid in the standardisation of how such characteristics are graded.^32^

### 4.3 Findings from clinical studies

To date, there have only been three randomised controlled trials (RCTs) comparing SU versus RU ureteroscopes. The first was a trial by Qi et al. in 126 patients.^33^ This study found no significant differences in any of the following outcomes: stone free rate (SFR) at 1 month, operation time, hospital stay or complications. The authors concluded SU to be non‐inferior.^33^ A secondary analysis evaluating patients with lower pole stones <20 mm (*n* = 49) found that outcomes were comparable for all the same variables with the exception of SFR, which favoured the SU group (85% vs. 58.33%, *p* < 0.05). More recently, Ali et al. recorded results from a randomised study of 242 patients.^28^ The main finding was significantly higher manoeuvrability with the SU ureteroscope (WiScope) and less limb fatigability but at the cost of lower image quality compared to the Flex‐Xc.^28^ Another RCT from China found no differences in patient outcomes when using the PU3022A; however, image quality was rated to be superior compared to the Flex‐X2.^34^ The latter may be expected given that the latter model is fibre‐optic as opposed to digital. Beyond these randomised studies, there have been several cohort studies published, which support the non‐inferiority of SU models in terms of outcomes such as SFR and hospital stay.^35^

### 4.4 Infection and postoperative complications

Reprocessing is a time intensive process that combines both manual and machine automated steps, and although protocols vary between sterile processing departments, drying alone can take over 3 h.^36^ Essential elements include pre‐cleaning, leak testing, manual cleaning, visual inspection, disinfection/sterilisation, storage and documentation. Based on the Spaulding classification, endoscopes can be categorised as semi‐critical devices with a requirement for high level disinfection.^37^ However, based on the functions that modern day ureteroscopes perform, they could also qualify as critical devices and therefore sterilisation should be performed as compulsory step and not just as a recommended supplement. SU ureteroscopes serve to eliminate both risk of cross contamination and save time. Although outbreaks related to cross‐infection are rare, they do occur. Legemate et al. collected pre‐use ureteroscope cultures across 489 procedures and found positive results in 12.1%.^38^ However, uropathogens were found in only 2.3%, and none of these cases experienced postoperative infection. Chang et al. reported an outbreak where 15 patients were affected.^39^ In a recent retrospective study of 991 patients, Unno et al. found the risk of postoperative urinary tract infection to be twofold less likely in patients who underwent URS with SU ureteroscope (6.5% vs. 11.9%, *p* = 0.018).^40^ A similar study by Mourmouris et al. recorded lower rate of post–intervention sepsis in patients in SU group.^41^ Although these results favour SU ureteroscopes, there remains a limited pool of data to be able to draw firm conclusions. Recent randomised trial recorded no difference in postoperative complications including serious adverse events between SU and RU ureteroscope use.^33^ Note that it is possible for SU ureteroscope to be contaminated prior to use; for example, if seal is damaged during delivery, users should inspect before use.

### 4.5 Cost

Expenditure associated with this technology is one of the main reasons for a slowed uptake across many parts of the world, especially those with less resources. Results of cost comparison studies reveal varying estimates.^20^, ^42^ This heterogeneity is largely because values used such as for acquisition costs, repair prices and scope longevity vary widely. For example, depending on the location and source, reported purchase prices for RU ureteroscope have varied between US$13 611 and US$85 000.^42^ Sterilisation costs are relatively comparable (≈US$100), and this covers equipment such as chemicals, brushes, personal protective items and the low temperature STERRAD cassette (≈US$20).^36^ In addition to this, there are the labour costs for reprocessing the equipment. The reported costs for repair rates range from US$2480 to US$7521 for RU scopes. The cost effectiveness of a ureteroscope is impacted by how durable it is, but the number of operations before repair varies from 8 to 29 cases.^43^ Overall, this results in cost per procedure varying between US$120 and US$1212 per procedure. Reporting of this metric is not uniform however, as durability of ureteroscope has also been reported in terms of operation hours as well as number of passes. It seems that there is no ‘one size fits all’ answer to whether it is financially profitable to switch over to SU ureteroscopes. Rather it comes down to the individual centre and it is therefore recommended for centres to perform their own micro cost analysis before deciding. Taguchi et al. did this at their institution and the authors found the overall costs for URF‐P6 (US$2799.72) and LithoVue (US$2852.29) to be comparable.^44^ Calculation models have been proposed to help centres assessing cost effectiveness based on their own data.^45^, ^46^ Ventimiglia et al. reported the implementation of a hybrid model in a high volume rather than compete conversion to SU models, which allowed for their use to be employed for select cases.^47^ This hybrid strategy resulted in prolonging the life cycle of RU ureteroscopes by 40%. High volume centres have recorded total repair costs per annum up to US$100 000.^16^ Martin et al. found that the threshold where the cost benefit favours RU URS was 99 cases.^48^ Complete conversion to the use of SU models is therefore more likely to be financially feasible in low volume centres that have limited reprocessing resources. They also offer a practical option in centres that use satellite smaller hospitals to operate day case surgery. Usawachintachit et al. performed a prospective case control study comparing SU and RU ureteroscopes and found that for stone removal cases, the mean difference was 13 min (70.3 vs. 57.3 min, *p* < 0.05) less in the SU group.^49^ The authors estimated that this would equate to savings of US$250.

A new development has been the updated coding reimbursement for SU device has been adapted in certain areas such as the Medicare Hospital Outpatient Prospective Payment System (OPPS). Given it fulfils requirements for an innovative device, it qualifies for a transitional pass‐through payment, which equates to an additional reimbursement.^50^ This system has already been established for other devices in urology such as for sacral neuromodulation stimulators (SMS).

### 4.6 Environmental impact

The healthcare sector currently accounts for 4.4% of global greenhouse gas emissions.^51^ One of the main concerns regarding SU ureteroscopes is the physical waste produced.^52^ SU endoscopes produce 4.1 times the volume of disposal waste compared to RU models (approx. 1 kg more per scope).^53^ SU endoscopes use more natural resources such as oil for non‐recycled plastic, which is one of the major components. However, it is worth noting that RU endoscopes also carry ecotoxic properties and a significant carbon footprint associated with use of chemicals (alcohol, detergents, disinfectants), clean water requirements (≈80 L per cycle per scope) and energy consumption (≈0.33 kWh per case).^52^, ^53^ Common to both SU and RU is the requirement for minerals to develop electronic components. There is also a significant CO_2_ footprint associated with transport of new endoscopes, those sent for repair and at the time of disposition. Factory locations for manufacture and repair are often located in another country.

Borofsky et al. reported their multi‐institutional pilot experience in the United States of a partnership with a medical waste company (Sharps Compliance Inc, Houston, TX) that aimed to salvage metal and electronic components while electricity was generated from steam energy generated during incineration of medical waste.^54^ This process led to 87% of the total physical waste being repurposed. However, while the latter can contribute to renewable energy, greenhouse gases (GHGs) production is sizeable and therefore deleterious. To date there has only been one study, which has specifically assessed the environmental life cycle of SU versus RU ureteroscopes.^55^ It concluded that, overall, the carbon footprint of LithoVue (4.43 kg) was comparable to Olympus VRF (4.47). However, considerations such as natural resources, GHG emissions from incineration and landfill waste were not included in the analysis. The majority of studies in the setting of bronchoscopes and duodenoscopes concluded that SU models still have a worse impact on the environment.^53^

## 5 ARGUMENTS FOR REUSABLE URETEROSCOPES

Although reported repair rates vary, it is known that surgeon experience and investment in the education of operational staff can affect longevity of RU ureteroscopes.^56^ This is especially relevant given that once the ureteroscope has been repaired for the first time, the time until next repair is considerably less.^57^ The variations in the size and shape of certain RU models still offer advantages not yet present in SU models (Table 2). This includes the 4.9 Fr tapered tip of the Olympus URF‐P7. The smaller size has been found to translate to clinical advantages especially for negotiating access to the ureteric orifice in more challenging anatomy such as children and pregnancy.^58^, ^59^ That model also has a smooth bullet shape to it that is lacking in SU models. Although some of these are tapered, inspection reveals the contour edges are less smooth. Use of accessories such as baskets is a known contributor to ureteroscope damage. Use of SU models are often put forward for complex cases at higher risk of scope breakage, for example, lower pole stone with steep infundibulopelvic angle or heavily encrusted stent. While this seems sensible, it could be argued that if a case is determined to be so high risk for scope damage, an alternative (e.g., miniaturised PCNL) is perhaps a more suitable option.^60^ Furthermore, benchside studies report scope degradation after use in simulator models and clinical studies have shown decreased function during use as well as need to convert to RU models during a case due to deterioration of image quality and deflection loss.^42^, ^61^ Sudden loss of image and device failure has also been reported with SU ureteroscopes; although this can also occur with RU ureteroscopes too.

**TABLE 2:** Advantages and disadvantages of single use ureteroscopes.

Advantages	Disadvantages
Reduced risk of contamination Potential for teaching novices Can use accessories with no concern for longevity of scope Use for cases with difficult anatomy and high risk for scope damage, for example, heavily encrusted stent Some parts likely able to be reprocessed in future Available with built–in accessories, for example, LithoVue™ Empower Functional properties match reusable scopes Cost effective in small volume centres and remote sites with no reprocessing facility. Newer models available that do not require specific image processor or monitor Future models to include pressure control, for example, LithoVue™ Elite	Cost benefit not yet known Plastic waste material More CO_2_ energy emissions with disposable equipment Greater mineral consumption Smaller tip dimensions available in re‐usable models Not available with NBI Reports of sudden image loss Reports of need to convert to reusable scope due to poor image More dependent on reliable outside supply Equipment failure not reimbursed by nearly all of companies Durability of instruments not fully known

Abbreviation: NBI, narrow band imaging.

Endoscopic combined intra‐renal surgery (ECIRS) is also argued as a scenario to consider SU models. However, a recent study found that use of accessories such as baskets rendered more damage to ureteroscopes than performing ECIRS.^62^ It may be that with laser advancements such as pulse modulation and improved dusting capabilities, requirement for basket use goes down and scopes maintained longer.

## 6 FUTURE PERSPECTIVES

Other SU endoscopes used in medical setting have seen high growth rates in recent years. Sales for SU bronchoscopes and rhinoscopes have increased at 124% and 441% per year.^63^ It seems likely that the trends in urology will also be upward. Borja Brugés et al. evaluated patient preferences on having SU or RU flexible cystoscope and found 88% of respondents to opt for the former.^64^ There are number of modifications and upgrades that are likely to occur over the coming decade and beyond. This includes introduction of wireless connection, suction and development of smaller image sensor chips allowing for reduction in tip dimensions.^65^ Overall, the product market price will likely go down as a result of competition and cheaper components. Once pressure monitoring is established, this could be coupled with temperature sensors. The next goal would be to have an automated control system to compensate for intra‐operative changes. It is likely that more parts will be able to be repurposed in the future, and although the scopes may not be RU, they may be recyclable to an extent.

## 7 CONCLUSION

SU ureteroscopes have favourable physical characteristics including modifications and low weight that translate to certain ergonomic advantages for the surgeon. Clinical outcomes match those of RU models. However, both the economic and environmental sustainability warrant further research. Further studies are also needed to evaluate if SU models result in lower infection rates and to determine durability and issue of device failure intra‐operatively.

## AUTHOR CONTRIBUTIONS

**Patrick Juliebø‐Jones:** Conception; data collection; analysis; writing of draft and revision. **Eugenio Ventimiglia:** Conception; data collection; editing and writing of manuscript. **Bhaskar K. Somani:** Data collection; editing and writing of manuscript; supervision. **Mathias Sørstrand Æsøy:** Data collection; editing and writing of manuscript. **Peder Gjengstø:** Data collection; editing and writing of manuscript. **Christian Beisland:** Conception; data collection; editing and writing of manuscript; supervision. **Øyvind Ulvik:** Conception; data collection; editing and writing of manuscript; supervision.

## CONFLICT OF INTEREST STATEMENT

Øyvind Ulvik has acted as a consultant for Olympus. The other authors have nil to declare.
